# Interactions between Soybean Trypsin Inhibitor and Chitosan in an Aqueous Solution

**DOI:** 10.3390/polym15071594

**Published:** 2023-03-23

**Authors:** Yihao Zhang, Ruijia Liu, He Li, You Li, Xinqi Liu

**Affiliations:** Beijing Advanced Innovation Center for Food Nutrition and Human Health, Beijing Engineering and Technology Research Center of Food Additives, National Soybean Processing Industry Technology Innovation Center, Beijing Technology and Business University, Beijing 100048, China

**Keywords:** soybean trypsin inhibitor, chitosan, electrostatic interactions, complex coacervations

## Abstract

Supramolecular structures obtained from protein–polysaccharide association may be applied to encapsulate bioactive compounds or to improve the physical stability and texture properties of colloid–based products. In this study, the interaction of 0.1 wt% soybean trypsin inhibitor (STI) with different concentrations of chitosan (CS) in aqueous solutions was investigated under different pH by the analysis of state diagram, turbidity, zeta potential, spectroscopy, and microstructure; the protective effect of STI–CS complex coacervates on STI stability in simulated gastric juice was also discussed. The results suggested that interactions between STI and CS could form soluble/insoluble complexes mainly through hydrophobic interactions (pH 4.0) or electrostatic interactions (pH 6.0). The CD spectra showed that the secondary structure of STI did not change significantly when CS with the same charge was mixed with STI, and the secondary structure of STI was slightly changed when CS with the opposite charge was mixed with STI. Simulated gastric digestion experiments showed that the complex formed by non-covalent bonding had a protective effect on the active protein. This study provides information about the effect of different CS concentrations and pH values on the formation of complexes of CS and STI in an aqueous solution and provides theoretical references for the construction of supramolecular-structured carrier substances based on CS and STI.

## 1. Introduction

Compared with traditional chemically synthesized drugs, protein and peptide drugs have high efficacy, low toxicity, and strong specificity, and are growing rapidly in the biopharmaceutical and nutraceutical markets [1,2,3]. By 2015, there were more than 80 kinds of protein and peptide drugs on the market all over the world [3]. However, the enzyme/pH sensitivity of peptides and proteins makes their oral bioavailability extremely low [1,4,5]. Currently, the strategies to improve the oral bioavailability of protein and peptide drugs include chemical modification (polyethylene glycol modification, glycosylation modification, small molecule coupling, and lipidation) and formulation strategies (combination of absorption enhancers, enzyme inhibitors, mucoadhesive agents, and carrier delivery), the last of which in formulation strategies shows superior properties and plays an important role in shielding protein and peptide structures from external environments (e.g., extreme pH and proteases), releasing slowly in a targeted manner and facilitating osmotic absorption [3].

Proteins and polysaccharides are common natural polymers found in food and are of great interest due to their biodegradability, high nutritional properties, and functional properties [6]. Proteins and polysaccharides can facilitate the formation of complex coacervates through covalent binding interactions, such as the one formed in the Maillard reaction, or through non-covalent interactions, including electrostatic, hydrogen bonding, and van der Waals interactions [6,7]. Protein–polysaccharide complex coacervates have properties such as improving the stability of active compounds, controlling their release, reducing volatile flavor loss, and masking undesirable tastes [6,8]. In addition, supramolecular complexes obtained by protein–polysaccharide binding can be used as delivery vehicles to encapsulate bioactive compounds. Thus, protein–polysaccharide interactions have potential applications in the food, cosmetic, and pharmaceutical industries [9].

Chitosan (CS) is a natural polysaccharide extracted from the shell of crustaceans or insects and is the second most widely spread in the world [10]. Structurally, CS is a macromolecular biopolymer composed of D-glucosamine and N-acetyl-D-glucosamine linked by β-(1-4)-glycosidic bonds, and the amino groups present in its molecular structure can acquire positive charges when dispersed in acidic media (pH < 6.4) [9]. In addition, CS is non-toxic, non-carcinogenic, and has some important properties such as biocompatibility, biodegradability, bio-adhesion, and pro-permeability [11,12]. Because of its outstanding material properties and permeation enhancement, CS-based encapsulation systems are considered to be one of the most promising matrices for protein or peptide encapsulation and delivery [13,14]. Studies have reported the inhibited and rapid release of CS encapsulated bovine serum albumin in the gastric (pH 1.2) and intestinal environments (pH 7.4), respectively [15], which is useful but still defective for the protection of peptides and protein-like functional components in the gastrointestinal tract. Currently, studies have tried to combine CS with other polymers to improve the properties of the delivery systems.

Soybean trypsin inhibitors (STI) are the main active proteins in soybeans, mainly including Kunitz and Bowman–Brik trypsin inhibitors [16], which are resistant to acid, alkali, heat, and pepsin hydrolysis, etc. [17]. They can block the activity of trypsin and chymotrypsin in the gastrointestinal system, protect against the hydrolysis of dietary functional proteins and peptides [18,19], and have anti-cancer, anti-inflammatory, anti-bacterial, and hypoglycemic functional properties [20,21,22]. Studies have shown that the coadministration of soybean trypsin inhibitor with teriparatide has a significant effect on the oral absorption of teriparatide [23]. Co-administration of insulin with a protease inhibitor and Ca^2+^ chelator decreases the hydrolysis of insulin by protease, elevates paracellular permeability, and improves the oral bioavailability of insulin [24].

At present, many studies have focused on the ability to form complex coacervates between inexpensive plant proteins with high nutritional value and polysaccharides as well as the mechanisms of formation, such as hemp isolate protein–gum Arabic [25], rapeseed isolate–gum Arabic [16], pea protein isolate–beet pectin [26], pea protein isolate–chitosan [27], and soy protein isolate–chitosan [28]. There are few studies on the formation of complexes of functionally active proteins with polysaccharides through non-covalent bonds. In this paper, we explored the interaction of macromolecule polymeric CS with small-molecule soybean trypsin inhibitors which can protect protein-polypeptide from protease degradation and also discussed the conformational changes of soybean trypsin inhibitor in the formation of complex coacervates. This study could provide a theoretical basis for constructing functional active STI–CS complex coacervates as potential carriers of proteins and peptides.

## 2. Materials and Methods

### 2.1. Feedstocks and Reagents

Cooled, defatted soybean meal was provided by the Yuwang Ecological Food Industry Co., Ltd. (Dezhou, China), which was crushed through a 100-mesh sieve and stored at 0~4 °C. CS (Mw = 100,000 Da, degree of deacetylation ≥ 80%) was purchased from Shanghai Macklin Biochemical Technology Co., Ltd. (Shanghai, China). All other chemicals were bought from Sinopharm Chemical Reagent Co., Ltd. (Shanghai, China). All materials were of analytical grade and chromatographically pure.

### 2.2. STI Preparation

STI preparation was performed according to previous procedures [29]. Briefly, the cooled, defatted soybean meal was added to deionized water at a solid/liquid ratio of 1:10 (*w/v*), adjusted to pH 7.5 with 1 M NaOH, extracted in a water bath at 50 °C for 50 min, and centrifuged at 4000 rpm for 20 min. The supernatant was collected, after which the precipitate was added with deionized water at a solid/liquid ratio of 1:4. This process was repeated once again, after which the subsequent supernatant was mixed with that obtained during the first procedure. The pooled supernatant was adjusted to pH 4.5 with 1 M HCl and centrifuged (4000 rpm, 20 min, 4 °C) to remove the protein isolate to obtain the soybean whey. The soybean whey was adjusted to pH 7.5 with 1 M NaOH, placed at room temperature for 60 min, and centrifuged (4000 rpm, 20 min, 4 °C) to remove the precipitate followed by adjusting to pH 4.0 with 1 M H_2_SO_4_. Ground ammonium sulfate powder was slowly added to the soybean whey until reaching a final saturation level of 40% to be stored at 4 °C for 120 min and centrifuged at 4000 rpm for 20 min. The protein precipitate was collected, dissolved in deionized water, adjusted to pH 7.5 with 1 M NaOH, and processed ultrasonically for 20 min. The precipitate was removed by centrifugation (4000 rpm, 20 min, 4 °C) while the supernatant was dialyzed overnight in a 200 Da dialysis bag (MYM biological technology company limited). At the end of dialysis, the supernatant was freeze dried as STI and stored at −20 °C. The purity and the specific activity of STI were 71.11% and 1442.5 TIU/mg, respectively.

### 2.3. Preparation of STI–CS Mixture System

CS and STI were accurately weighed and dissolved in 1.0% (*v/v*) acetic acid and deionized water, respectively. The samples were magnetically stirred for 2 h at 25 °C and placed overnight at 4 °C to moisten the sample thoroughly. The samples were then processed ultrasonically for 20 min to remove the bubbles and stored at 4 °C for later use. The CS and STI solutions were separated into 5 groups and mixed with different mass ratios as shown in Table 1.

### 2.4. Phase Diagram

The pH of 0.1 wt% CS, 0.1 wt% STI, and the mixed system (SC–0.01, SC–0.05, SC–0.10, SC–0.15, and SC–0.20) as shown in Table 1 was accurately adjusted to 3.0, 3.5, 4.0, 4.5, 5.0, 5.5, 6.0, 6.5, 7.0, 7.5, 8.0, 8.5, 9.0, 9.5, and 10.0, respectively by using 0.1, 0.5, or 1.0 M HCl or NaOH. Then, the samples were left for 30 min at 25 °C. A state diagram from mixtures as a function of CS concentration and pH was constructed from visual observations.

### 2.5. Determination of Turbidity [30]

For the determination of turbidity, 0.1 wt% CS, 0.1 wt% STI, and the mixed systems (SC–0.01, SC–0.05, SC–0.10, SC–0.15, and SC–0.20) were adjusted to different values of pH (3.0, 3.5, 4.0, 4.5, 5.0, 5.5, 6.0, 6.5, 7.0, 7.5, 8.0, 8.5, 9.0, 9.5, and 10.0), respectively and left for 30 min at 25 °C. The samples were poured into a 1 cm path length cell and the absorption value of each sample was measured using a UV-visible spectrophotometer (Agilent Technologies Cary 60, Santa Clara, CA, USA) at 600 nm. All measurements were performed in triplicate at room temperature.

### 2.6. Determination of Zeta Potential

To evaluate the overall surface charge, the zeta potential (ζ, mV) of STI, CS, and their mixed system was determined using a Zetasizer Nano ZS90 apparatus (Malvern Instruments, Malvern, UK) at pH values of 3.0~10.0. The samples were diluted (5–fold) in sodium acetate buffer solution to avoid multiple scattering and measured in triplicate at room temperature [25,31]. The refractive indexes of the protein, CS, and their complex were 1.42, 1.7, and 1.55, respectively.

### 2.7. Fluorescence Spectroscopy

Fluorescence measurements were performed at 25 °C using an FS5 fluorescence spectrometer (Edinburgh Instruments, Livingston, UK) equipped with a 1 cm path length quartz cell. The excitation wavelength was 280 nm, and the emission wavelength was recorded between 290 and 500 nm with excitation and emission slit widths set at 5 nm. The prepared samples were subjected to a 5-fold dilution using the acetic acid–sodium acetate buffer with the same pH to measure fluorescence [32].

### 2.8. Far-UV Circular Dichroism (CD) Spectroscopy

Far-UV CD measurements of STI solutions and STI–CS mixtures at different pH (4.0, 6.0) were performed using a dichrograph instrument (MOS–500, BioLogic, Seyssinet-Pariset, France), equipped with a quartz cuvette of 0.1 cm light-path length [25]. All far-UV CD spectra (190~250) were obtained in a nitrogen atmosphere at 25 °C. The spectra were analyzed via https://bestsel.elte.hu/index.php (accessed on 7 September 2022) to calculate the α-helix, β-fold, β-turn, and random curl percentages.

### 2.9. Fourier Transform Infrared (FTIR) Spectroscopy [33]

The stock solutions of STI, CS, and the aggregates of SC–0.10 were freeze dried, 2 mg of which was ground thoroughly with potassium bromide in an agate mortar, placed in a tablet press, and pressed into thin slices. During the test, the background was first collected followed with the infrared spectrum of the sample. The FTIR (Thermo Scientific Nicolet iS20, Waltham, MA, USA) spectra were recorded with an average of 32 scans from 4000 to 400 cm^−1^ at a resolution of 4 cm^−1^.

### 2.10. Microstructure Analysis

The microstructures of STI, CS, and STI–CS samples were observed using the field-emission scanning electron microscope (Zeiss Merlin Compact, Jena, Germany). The freeze-dried samples were fixed to the sample stage using conductive double-sided tape and sprayed with gold for 45 s using an Oxford Quorum SC7620 sputter coater, followed by a Zeiss Merlin Compact scanning electron microscope being used to photograph the sample morphology with an accelerating voltage of 3 kV during morphology photography.

### 2.11. Examination of the STI–CS Stability

Procedures were modified from the previous study [29]. Briefly, 60 mL STI–CS (SC–0.10) was added into 60 mL of simulated gastric juice (0.2 g/100 mL NaCl, 0.32 g/100 mL pepsin, and HCl adjusted to pH 1.5), pre-warmed at 37 °C, and incubated in a water bath shaker. Samples were taken at 5 min, 10 min, 15 min, 20 min, 25 min, 30 min, 40 min, 50 min, 60 min, 80 min, 100 min, and 120 min, respectively, followed by neutralization by an equal volume of 0.1 M NaOH to determine the trypsin inhibitory activity with STI as control.

### 2.12. Statistical Analysis

SPSS Statistics 19 software (IBM, Chicago, IL, USA) was used for data processing. The experimental data were expressed as “X ± SD”. Significant differences between measurements were set at *p* < 0.05. Origin 2021 was used for data visualization.

## 3. Results and Discussion

### 3.1. Phase Diagram of CS–STI Mixed Dispersions

**Figure 1:**
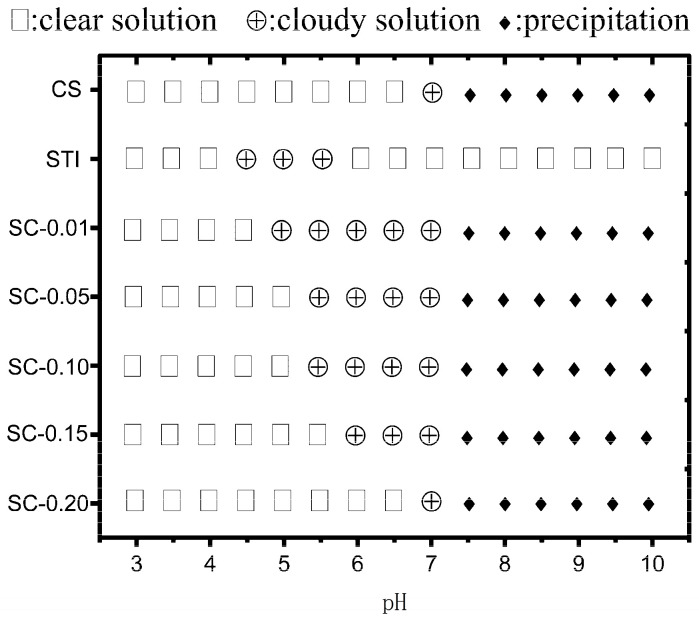
Phase behavior of STI, CS, and mixed systems (SC–0.01, SC–0.05, SC–0.10, SC–0.15, SC–0.20) with pH variation.

Figure 1 shows the effects of pH on the phase behavior of STI, CS, and STI-CS mixed dispersions. In an aqueous CS solution, when the value of pH was less than 7.0, the CS solution was clear and transparent, and when pH reached 7.0, the solution became cloudy. This relates to the literature report stating that CS’s pKa is between pH 6.3 and 7.2 [34]. When the pH of the CS solution is less than 6.5, the amino groups of CS molecules are protonated, and CS could be completely dissolved in water. While the solution pH is close to pKa, the repulsive force between CS molecules is weakened, and the intermolecular aggregation of CS occurs, resulting in turbidity of the dispersed solution [35]. As the pH value continued to increase, the CS solution precipitated due to its inability to dissolve in alkaline conditions. When the pH of the STI aqueous solution was less than 4.5 or more than 5.5, the solution was clear and transparent, which became cloudy when the pH was in the range of 4.5 to 5.5. This phenomenon may be attributed to STI’s isoelectric point of around 4.5. At the isoelectric point of the protein, the surface static charge of the amphoteric molecules was zero, the repulsive force between protein molecules was weakened, and aggregation occurred between protein molecules, which can easily form turbidity or precipitation [36,37]. The phase state of the mixed system (SC–0.01, SC–0.05, SC–0.10, SC–0.15, SC–0.20) was different from that of STI. The comparison of the mixed system with STI dispersion at pH 4.5, which exhibited a clear solution, implies that the CS interacted with the STI, changed the STI properties, and affected the self-aggregation of the STI molecules. As the concentration of CS increased, the pH at which the mixed system became cloudy gradually increased. When the CS concentration elevated to 0.2% (*w/v*), STI was very stable under acidic conditions (pH < 7.0), while the solution was clear and transparent, and the formation of STI self-aggregation was inhibited, which was conducive to STI application in acidic conditions. When the CS concentration dropped to 0.01% (*w/v*), the solution of SC–0.05 became cloudy between pH 5.0 and pH 7.0, which further indicates that the addition of CS affected the phase behavior and helped to form complex coacervates at the same time.

### 3.2. Turbidimetric Analysis

**Figure 2:**
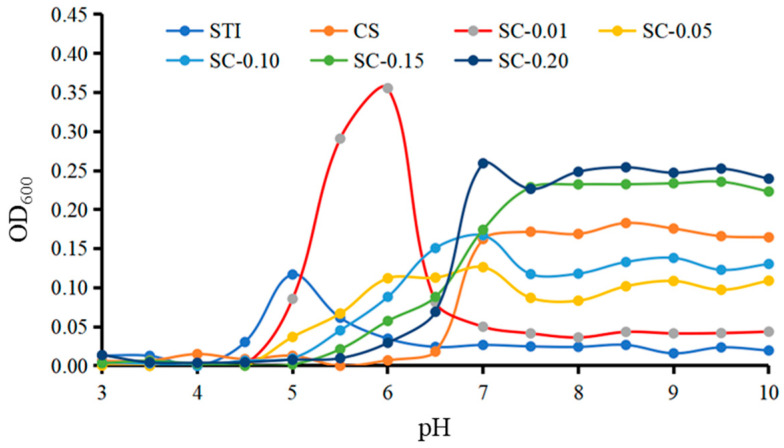
The turbidity changes of STI, CS, and mixed systems (SC–0.01, SC–0.05, SC–0.10, SC–0.15, SC–0.20) over different pHs.

**Figure 3:**
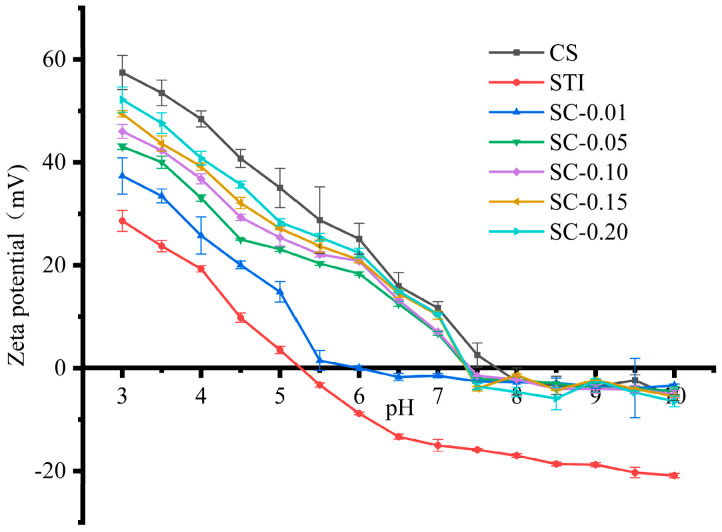
The zeta potential changes of STI, CS, and the mixed systems (code: SC−0.01, SC−0.05, SC−0.10, SC−0.15, SC−0.20) over different pHs.

Figure 2 shows the turbidity of CS, STI, and mixed systems (code: SC–0.01, SC–0.05, SC–0.10, SC–0.15, SC–0.20) at different pHs. The turbidity of CS was low in the pH range of 3.0 to 6.5. When the value of pH was greater than 7.0, the turbidity increased rapidly, forming insoluble aggregates and causing light scattering. This is consistent with the previous results suggesting that turbidity values of CS solutions increase after pH exceeds 7.0 [38]. For the aqueous STI solution, the turbidity was low when the pH was less than 4.0 or greater than 6.5. As the value of pH increased from 4.0 to 5.0, the turbidity raised rapidly and reached a maximum at pH 5.0. This may be because of the fact that when the solution is at pH 5.0, the aqueous STI solution is exactly near the isoelectric point. At the isoelectric point, protein molecules carry less or no charge, and the electrostatic force between molecules is weak, resulting in protein–protein intermolecular aggregation and the value of OD_600_ being maximum [12]. The speculation is consistent with the results shown in Figure 3. With the continuous pH elevation, the ionization degree of amphoteric electrolyte, the amount of negative charge of protein, and the electrostatic repulsion increased while the protein aggregates depolymerized and the turbidity value decreased. The turbidity value of the STI solution approached zero when the pH was in the range of 6.5 to 10.0. The turbidities of the mixed systems (SC–0.01, SC–0.05, SC–0.10, SC–0.15, SC–0.20) were different from those of STI and CS in the range of pH 4.0 to 7.0, respectively, which indicated an intermolecular interaction between STI and CS. The cloudy solution’s pH changed from 4.0 (STI) to 4.5 (SC–0.01, SC–0.05), 5.0 (SC–0.10, SC–0.15), and even 5.5 (SC–0.20). The turbidity of STI was the highest at pH 5.0, but the turbidity of the mixed system was lower than that of STI at pH 5.0, which suggests CS could stabilize STI, prevent STI from self-aggregation, and improve STI instability in an acidic environment. Similar results were found in β-lactoglobulin/CS complexes, myofibrillar protein/CS complexes, casein/CS complexes, and pea protein isolate/CS complexes [12,39,40,41]. When the pH of the mixed system was greater than 6.0, the turbidity of the mixed system was higher than the individual solution, indicating that protein and CS formed insoluble complexes.

The turbidity of mixed systems (SC–0.01, SC–0.05, SC–0.10, SC–0.15, SC–0.20) varied at different CS concentrations. At pH 5.5, the turbidity of CH–0.01 was larger than STI, while the turbidity of other mixed systems was smaller than STI. This may be because of the low concentration of CS in SC–0.01 and the low charges carried by CS, which neutralized with STI and the charge of the complexes tended to zero, resulting in the formation of the insoluble complexes. While the concentration of CS in SC–0.05, SC–0.10, SC–0.15, and SC–0.20 became higher, CS carried a larger number of charges. After intermolecular interactions with STI through electrostatic force, the complexes behaved as the same type of positive charge as CS, and the occurrence of mutual repulsion formed soluble complexes. The results showed that higher CS concentrations were beneficial to the formation of electrostatic complexes and improved the stability of STI in less acidic conditions of pH 4.0~pH 6.0. The lower CS concentration may reduce the stability of STI and accelerate the formation of STI–CS complexes.

### 3.3. Zeta Potential Measurements

Previous studies have shown that the electrostatic interactions between oppositely charged macromolecules constitute the main driving force to form protein–polysaccharide complexes and coacervates [42,43]. The zeta potential was measured to understand the effects of pH and CS concentration within mixtures on the electrostatic interactions between STI and CS [32]. As shown in Figure 3, the zeta potential of STI decreased from positive to negative values (from 28.63 ± 2.04 to −20.09 ± 0.43 mV) with pH from 3.0 to 10.0 because of the deprotonation of the amino and carboxyl groups carried by STI. The STI had a crossover point at around pH 5.2, where the zeta potential was zero mV, and was considered an isoelectric point (IEP). At this time, the STI solution was prone to precipitate, which confirmed that the turbidity of the STI solution reached its maximum at pH 5.0 (Figure 2). The zeta potential of CS also decreased from 57.47 ± 3.30 mV to −5.71 ± 0.29 mV with the increasing of pH from 3.0.0 to 10.0. CS dispersions displayed a positively charged polyelectrolyte behavior at pH 3.0 to 7.5, which is commonly attributed to the protonation of the glucosamine segments to form ammonium groups (–NH^3+^) [44]. CS dispersions displayed a negatively charged polyelectrolyte behavior at pH 8.0 to 10.0, and the zeta potential was close to zero mV. The zeta potential of CS decreased with the increase in the value of pH. The reduction in the zeta potential of CS could weaken the repulsion forces among individual molecules, resulting in the formation of large-sized aggregates with the turbidity being increased, which is consistent with the CS turbidity curves from Figure 2.

The mixtures’ zeta potential was reduced with a decrease in CS concentration or increase of mixtures pH and was found between STI and CS dispersions. In the range of pH 3.0 to 5.0, the zeta potential of the mixed systems at different CS concentrations all showed more positive charges than STI, indicating that the addition of CS improved the solubility and stability of STI in low-acidity conditions (pH < 5.0). In the range of pH 5.0 to 7.0, the zeta potential of SC–0.01 was around zero mV, which was lower than that of STI or CS and may be because of the positive and negative electric neutralization as well as the formation of supramolecular composite condensate, and led to the decrease of its zeta potential. In addition, the zeta potential values of SC–0.05, SC–0.10, SC–0.15, and SC–0.20 were all positive, while STI alone showed negative values, which may be because of the complex formation between STI and CS, forming a supramolecular structure with CS as a shell and STI as a core. The zeta potential of STI–CS mixtures was increased as the CS concentration increased because of the positive zeta potential of CS molecules. However, in the range of pH 7.5 to 10.0, we observed that the curves of the mixed system overlapped with the curves of the CS, while the STI showed a larger negative value of zeta potential (Figure 3). This situation may occur because the formed complex does not release the STI rapidly as the pH increases, while the excess CS will form a precipitate under alkaline conditions, resulting in its zeta potential being consistent with that of mixed systems.

In the range of pH 5.0 to 6.5, compared with the results in Figure 2 and Figure 3, it could be seen that zeta potential is directly related to turbidity. At pH 6.0, the zeta potential of SC–0.01 was zero mV. At this time, the pH value was considered as the pKa value of SC–0.01. The intermolecular interactions between STI and CS occurred, and the net charge was zero mV. While the intermolecular repulsion was the weakest and mutually aggregated, the turbidity value was the largest. With the increase of CS concentration, the zeta potential value of the mixed system was larger than that of STI, implying that the intermolecular repulsion of the mixture was larger than STI and the stability was better than STI, which is consistent with the turbidity measurement.

The results here suggest that electrostatic interaction between STI and CS could occur in the range of pH 5.2 to 7.5, and core shell supramolecular complexes are formed. This has theoretical guidance for the encapsulation, delivery, and release of active proteins and peptides.

### 3.4. Fluorescence Spectroscopy

**Figure 4:**
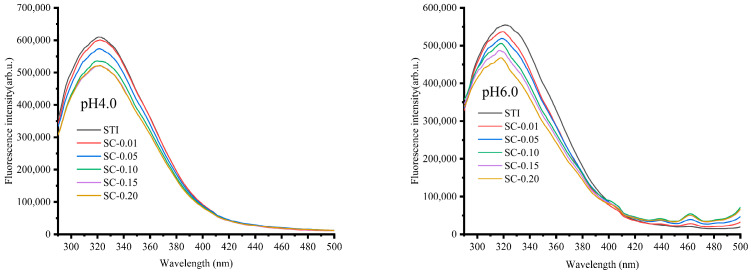
The effect of CS concentration on the fluorescence intensity of STI at pH 4.0 and 6.0.

The change of intrinsic fluorescence intensity after the interaction between STI and CS at different pH values was studied by fluorescence spectroscopy. Figure 4 shows the emission spectra of STI (excited at 280 nm) in the presence or absence of CS at pH 4.0 and 6.0. The spectrum of STI was characterized by a single peak with a maximum fluorescence wavelength (λmax) at around 321 nm. It is noteworthy that the fluorescence emission intensity of STI was higher at pH 4.0 than pH 6.0, which may be because of the STI at pH 6.0 having weaker intermolecular repulsion and stronger self-aggregation than that at pH 4.0, and more fluorescent groups being wrapped, resulting in weaker fluorescence emission intensity [9]. At pH 4.0, with the increase of CS (no intrinsic fluorophore) concentration, the fluorescence emission spectrum of STI changed significantly, indicating that there is a certain degree of interaction between CS and STI molecules. The fluorescence emission intensity decreased gradually with the increase of CS, but the maximum emission wavelength of STI remained unchanged, indicating that the presence of CS has no obvious effect on the structural conformation of STI. At pH 6.0, the addition of CS also affected the fluorescence emission intensity of STI. With the increase of CS (no intrinsic fluorophore) concentration, the emission intensity of STI intrinsic fluorophore gradually decreased, indicating the occurrence of interaction between CS and STI molecules at pH 6.0. Unlike at pH 4.0, the maximum emission wavelength (λmax) of STI was blue-shifted as the CS concentration was increased at pH 6.0 with the peak position shifting to 319 nm. The result suggests that CS affects the structural conformation of STI and the interaction between STI and CS may form the CS–STI complex at both pH values via interaction. The fluorescence quenching phenomenon of STI could be attributed to the presence of inter and intramolecular interactions among the protein and polysaccharide chains. The intrinsic fluorophore of STI is surrounded by CS macromolecules, which inhibit STI fluorescence emission. Furthermore, CS is a polysaccharide with an NH^3+^ group (at pH < pKa = 6.4), and, together with other electron-deficient groups such as protonated histidine, lysine, and –COOH, may provoke the quenching of tryptophan residues [9]. The biological macromolecules are positively charged and the most important interaction types may be hydrophobic interaction and H bonding at pH 4.0 in the STI–CS complex. However, biological macromolecules carry heterogeneous charges and the formation of supramolecular structures may be driven by electrostatic interaction and stabilized by the H bond at pH 6.0.

### 3.5. Circular Dichroism Spectroscopy

**Figure 5:**
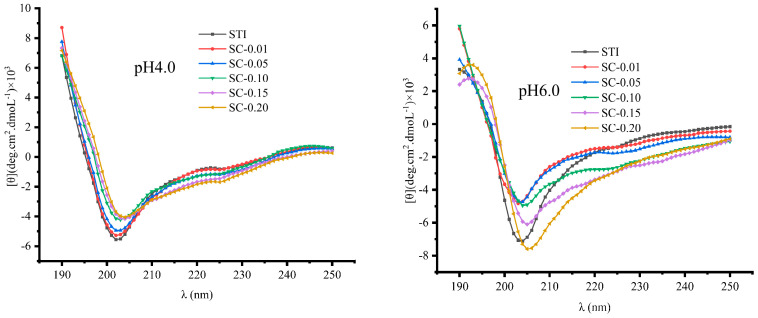
The effect of CS concentration on the CD spectra of STI at pH 4.0 and 6.0.

CD spectrum is a method for determining the secondary structure of proteins and is a reliable indicator of the global folding and unfolding process of the protein [45]. In this study, the spectra of STI, in the absence and in the presence of CS, were recorded in the range of 190 to 250 nm in order to evaluate if there were any changes in the secondary structure of the protein with the increase in CS concentration. Figure 5 shows the variation of CD spectra of mixed systems (code: SC–0.01, SC–0.05, SC–0.10, SC–0.15, SC–0.20) at pH 4.0 and pH 6.0 conditions, which were quantitatively analyzed to estimate the amount of the secondary structures in STI (Table 2). At pH 4.0, the deconvolution showed that the natural STI did not contain α-helix but mainly consisted of β and random coil structures, with β-sheet (42.07%), β-turn (14.45%), and random coil (43.48%). However, at pH 6.0, the secondary structures of STI had 38.67% β-sheet, 14.06% β-turn, and 47.20% random coil. The variation of pH from 4.0 to 6.0 caused a difference in the proportion of the secondary structures in STI. When the pH increased from 4.0 to 6.0, some carboxylate in amino acid residues was deprotonated and the net charge of the protein became negative. The intermolecular electrostatic repulsion of the STI molecules was weakened (zeta potential: from 19.30 ± 0.61 to −8.80 ± 0.26 mV), which could be responsible for altering the secondary conformation of the protein. Therefore, our results suggest that the pH affected intermolecular electrostatic repulsion, which was the primary type of interaction that stabilized secondary structures. The random coil increased and the β-sheet reduced, which reflected the STI as being more unstable. At pH 4.0, the addition of CS had a slight change on the CD spectrogram of STI. When the CS concentration was lower than 0.1%, the CD spectrogram of the hybrid system was consistent with that of STI. When the CS concentration was higher than 0.1%, the CD spectral intensity of the mixed system was slightly weaker than that STI, which may be because of the hydrophobic interaction between CS and STI, leading to a local decrease in STI concentration as well as CD intensity. As can be seen from Table 2, the ratio of the STI secondary structure in the mixed system at pH 4.0 was not significantly different from the ratio of the single STI secondary structure (*p* > 0.05), indicating that the CS concentration has no significant effect on the STI secondary structure at pH 4.0. The hydrophobic effect was not sufficient to change the secondary conformation of STI, which is consistent with the results postulated by the fluorescence above. At pH 6.0, the addition of CS had a significant effect on the CD spectra of STI, with CS in the range of 0.01~0.20%. The lower the CS concentration, the more obvious the effect on STI, which may be because of the fact that the positive charge carried by the low concentration of CS was exactly equal to the negative charge carried by STI, and stronger electrostatic adsorption occurred, generating a larger complex condensate, which precipitated out from the system, resulted in a decrease in the intensity of the STI spectrum of the mixed system, and was consistent with the turbidity value (Table 2). The secondary structure ratio of STI in the mixed system at pH 6.0 was significantly different from that of single STI (*p* < 0.05), which indicates that the addition of CS affected the secondary structure of STI at pH 6.0, probably because of the stronger electrostatic force which rearranged the spatial structure of STI and thus affected the secondary structure of STI.

### 3.6. Fourier Transform Infrared Spectroscopy

**Figure 6:**
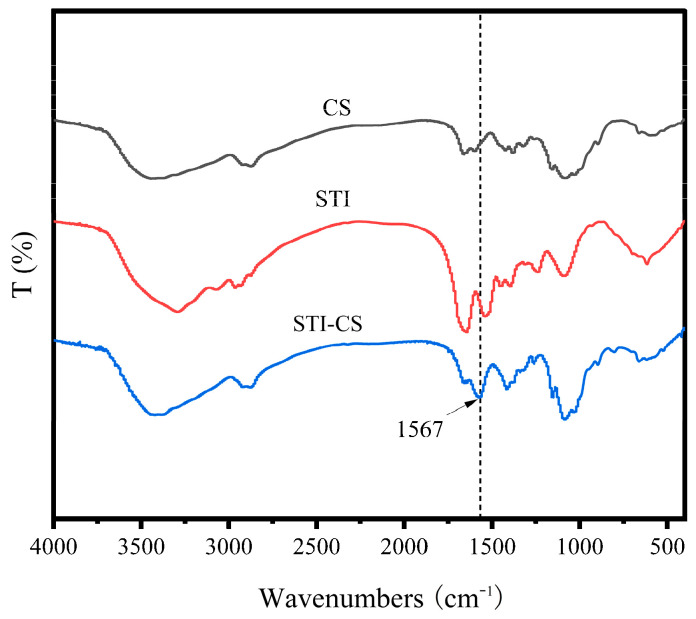
Fourier infrared spectra of CS, STI, and STI–CS (SC–0.10, pH 6.0).

The FTIR spectra of STI, CS, and STI–CS (SC–0.10, pH 6.0) are shown in Figure 6. The FTIR spectra of STI showed that there was an absorption peak of 1644 cm^−1^ related to C=O stretching vibration in the amide I band (1600~1700 cm^−1^). Additionally, the absorption peak at 1235 cm^−1^ appeared in amide III bands (1220~1330 cm^−1^) and was attributable to C–N stretching and N–H bending [28]. The FTIR spectrum of CS showed a strong amino characteristic peak at around 3431 cm^−1^. Other absorption peaks were also observed at 2869 cm^−1^ corresponding to the stretching vibration of the C–H bond; at 1655 cm^−1^ corresponding to the characteristic amide I band generated by the C=O vibration of the acetylation unit; at 1597 cm^−1^ corresponding to the absorption peak related to the NH^3+^ group; at 1405 cm^−1^ corresponding to the vibration of –OH and –CH; at 1157 cm^−1^ corresponding to the symmetrical tension of C–O–C; and at 1080 cm^−1^ relating to the C–O tensile vibration. The FTIR spectrum of STI–CS showed that there was no absorption peak at 1644 cm^−1^ and 1597 cm^−1^, but a new absorption peak at 1567 cm^−1^. It also showed that the complex coacervates between the two biopolymers of CS and STI changed the absorption peak of the carbonyl group of STI and the amino group region of CS, which may be because of the electrostatic interaction between the amino group of CS and the carboxyl group of STI or the formation of a new amide bond between STI and CS. The results of the infrared spectrum showed that CS was coupled with the soybean trypsin inhibitor through an amide bond.

### 3.7. Morphological Characteristics of STI–CS Complex

**Figure 7:**
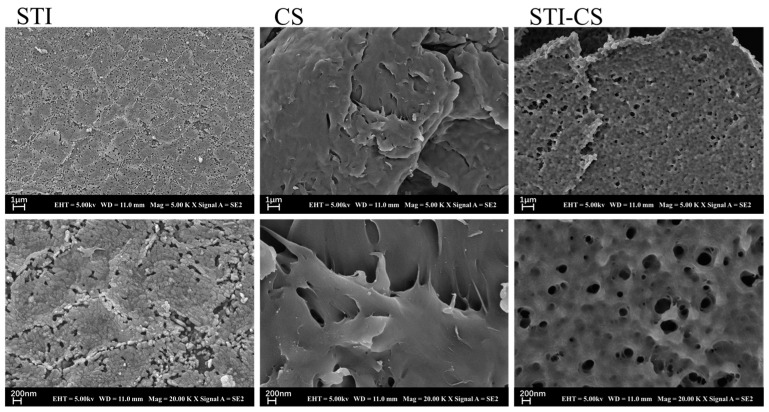
SEM micrographs of freeze-dried STI, CS, and STI–CS (SC–0.10, pH 6.0).

As shown in Figure 7, the surface structure of STI was composed of spherical individuals of uniform and tight arrangement. The surface structure of CS was smooth and irregularly “paste-like”. The lyophilized sample of the CS–STI complex had a different surface structure from both CS and STI, with a reticulated configuration that resembled a viscous paste (CS) covering the surface of small spheres (STI) and a very consistent distribution of mesh pores, with a size of about 200 nm, which is consistent with whey protein–chitosan or whey protein–xanthan gum complex studies and indicates that STI and CS could also form a complex with CS as the shell and STI as the core at pH 6.0 [31,46].

### 3.8. The Protective Effect of STI–CS Complex Coacervates on STI Stability

**Figure 8:**
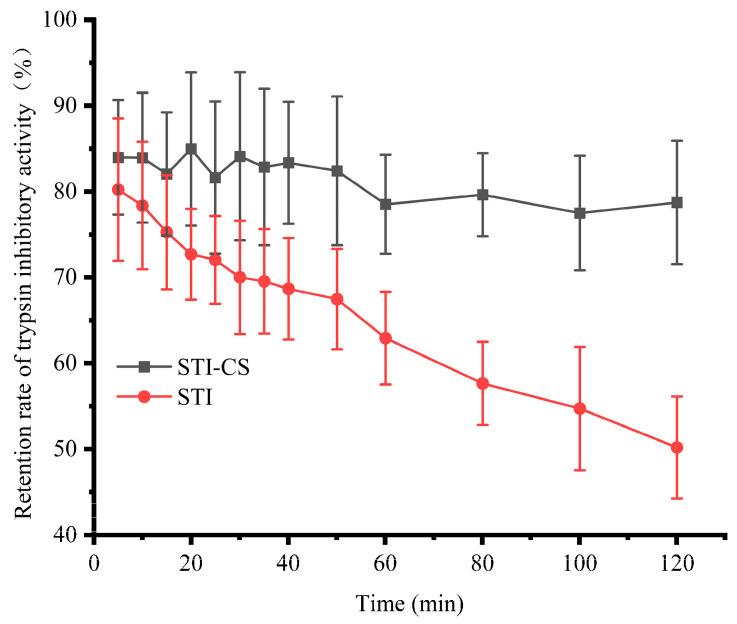
The effect of CS complex coacervates on STI stability.

Figure 8 shows the effect of complex coacervates (CS–STI) on STI stability in simulated gastric juice. Compared with free STI, CS–STI showed excellent stability in the presence of pepsin. After 50 min, the retention of trypsin inhibitory activity of STI was 67.47%. However, the retention of trypsin inhibitory activity of CS–STI was more than 82.41%. After 120 min, the retention of trypsin inhibitory activity of STI was 50.18%, but the retention of trypsin inhibitory activity of STI–CS was 78.73%. These results indicate that the formation of CS–STI complex coacervates was an effective approach to protect the STI activity. CS could improve the stability of STI in simulated gastric juice.

## 4. Conclusions

In this work, optical and spectroscopic results were linked to provide insight into the interaction between STI and CS through phase diagram drawing, turbidity, and potentiometric measurement. The effects of the CS concentration and pH on STI and CS molecule interaction were also discussed to more intuitively understand the underlying mechanisms. The combination of fluorescence, circular dichroism, and Fourier transform infrared spectroscopy was used to further understand the interaction between STI and CS from the perspective of protein molecular structure and investigate whether the interaction between STI and CS changed the structure of STI. The obtained results showed that the addition of the polymeric polymorph of CS changed the phase behavior and turbidity value of the mixed system and improved the stability of STI under acidic conditions, serving as theoretical guidance for the construction and optimization of STI–CS supramolecular structures for the encapsulation of protein and peptide bioactive substances. In vivo and in vitro studies on the physio-chemical functions of STI–CS complex coacervates in different matrices are warranted.

## Figures and Tables

**Table 1:** Composition of STI–CS system.

Code	The Solid Content in the Mixed Aqueous Solution	
	STI (wt%)	CS (wt%)
SC–0.01	0.10	0.01
SC–0.05	0.10	0.05
SC–0.10	0.10	0.10
SC–0.15	0.10	0.15
SC–0.20	0.10	0.20

**Table 2:** The effect of CS concentration on the secondary structure of STI.

Sample	pH 4.0				pH 6.0			
	α-Helix	β-Sheet	β-Turn	Random Coil	α-Helix	β-Sheet	β-Turn	Random Coil
STI	0	42.07 ± 1.65	14.45 ± 0.40	43.48 ± 2.01	0	38.67 ± 0.29 ^cd^	14.06 ± 0.33 ^bc^	47.20 ± 0.55 ^bc^
CH−0.01	0	42.50 ± 2.33	14.55 ± 0.83	42.85 ± 3.29	0	42.66 ± 0.59 ^a^	15.06 ± 0.04 ^a^	42.27 ± 0.62 ^d^
CH–0.05	0	41.90 ± 2.02	14.43 ± 0.93	43.68 ± 2.93	0	40.36 ± 0.04 ^bc^	14.53 ± 0.39 ^ab^	45.46 ± 0.23 ^c^
CH–0.10	0	42.13 ± 1.94	14.60 ± 0.55	43.25 ± 2.48	0	40.83 ± 0.04 ^ab^	13.97 ± 0.40 ^bc^	45.20 ± 0.39 ^c^
CH–0.15	0	41.73 ± 1.17	14.08 ± 0.37	44.18 ± 1.56	0	36.50 ± 1.48 ^e^	13.13 ± 0.53 ^c^	50.33 ± 1.96 ^a^
CH–0.20	0	40.08 ± 2.07	13.73 ± 0.78	46.20 ± 2.84	0	36.87 ± 0.78 ^de^	13.70 ± 1.12 ^bc^	49.43 ± 0.75 ^ab^

Results are presented as mean values ± standard deviation (n = 3). The data in each column are not significantly different (*p* > 0.05) (pH 4.0). Different letters on the same column indicate significant differences (pH 6.0).

## Data Availability

The data presented in this study are available on request from the corresponding author.
